# A dataset of gameplay videos with frame-wise board annotations, piece annotations, and state mappings for the Connect Four game

**DOI:** 10.1016/j.dib.2026.112553

**Published:** 2026-02-06

**Authors:** Pankaj Kumar G, Anitha M L, Arun Kumar M N

**Affiliations:** a PET Research Centre, PES College of Engineering, Visvesvaraya Technological University, Belagavi 590018, India; b Department of Computer Science and Engineering, Federal Institute of Science And Technology, Ernakulam, India; c Department of Computer Science and Engineering, PES College of Engineering, Mandya, Visvesvaraya Technological University, Belagavi 590018, India

**Keywords:** Board state representation, Move prediction, Image vectorization, Object detection, Game AI, Computer vision

## Abstract

This article describes a dataset derived from real-world video recordings of the physical board game Connect Four, focusing on visual perception and structured state representation. The dataset includes gameplay videos and frame-wise annotations obtained from human-versus-human matches played on a standard physical board. A total of 52 recorded games were selected for detailed annotation, from which board-level annotations, individual piece annotations, and image-based vector representations of game states were generated.

All visual and symbolic data components originate from annotated human gameplay recordings. An annotation program was developed to convert video frames into structured board matrices and corresponding vectorized representations. The dataset is organized into multiple components, including raw videos, annotated board regions, annotated piece locations, and vectorized board states.

In addition, an auxiliary dataset of one million unique playable board positions with associated move labels was generated computationally as a downstream resource. This auxiliary dataset is independent of the video data and is not required for the reuse of the computer vision components. The dataset can be reused for tasks involving board state extraction from images, visual-to-symbolic mapping, and downstream state–action modeling once visual states have been inferred.

Specifications Table

**Table:** 

Subject	Computer Sciences
Specific subject area	GameAI
Type of data	Video files (MP4); Image files (JPG); Tabular data (CSV); Text annotation files (TXT) Raw, Analyzed, Processed
Data collection	Data were collected from 52 human-versus-human Connect Four games recorded using smartphone cameras. Video frames were extracted and manually annotated at board and piece levels using Label Studio and a custom Python-based annotation tool. Board matrices and flattened 42-element vectors were generated from annotated frames. An auxiliary optimal-move dataset was generated independently through computational self-play by producing one million unique playable board states and assigning move labels using a Connect Four engine and minimax search. Only complete games with clear visual visibility were included in the video dataset.
Data source location	Data were generated and processed at the Department of Computer Science & Engineering, Federal Institute of Science And Technology, Ernakulam, Kerala, India.
Data accessibility	Repository name: Multimodal Dataset for Connect Four (Zenodo) [1] Data identification number: 10.5281/zenodo.17578931 Direct URL to data: https://zenodo.org/records/17578932 Instructions for accessing these data: The dataset is publicly available and can be accessed directly via the provided Zenodo link without restrictions.
Related research article	None

## 1 Value of the Data

• The dataset provides annotated video recordings of physical Connect Four gameplay, offering real-world visual data of structured tabletop interactions. These data are valuable for research on visual perception in constrained, grid-based environments and for studying board-game scenarios captured in real-world settings. The inclusion of frame-wise board and piece annotations supports the reuse of computer vision tasks, such as board detection, object localization, color-based piece identification, and image-to-state mapping in structured environments.
• Frame-wise board and piece annotations allow reuse for supervised computer vision tasks such as board extraction, disc localization, color-based classification, and object tracking under varying lighting conditions, occlusions, and camera viewpoints.The optimal move dataset, consisting of one million unique playable board positions with corresponding move labels, can be reused independently for studies involving game solving, state–action datasets, or algorithm validation in deterministic turn-based games.
• Annotated frames aligned with vectorized board-state representations enable reuse for machine learning and deep learning models that perform image-to-state mapping and visual-to-symbolic conversion from real-world images.
• The raw gameplay videos, independent of annotations, can be reused for unsupervised or weakly supervised video analysis tasks, including temporal segmentation, gameplay summarization, highlight extraction, and motion pattern discovery.
• An auxiliary optimal-move dataset is provided as a separate downstream resource intended for use after visual state extraction. It can be reused for supervised learning or benchmarking of state–action models once board states have been inferred from visual input, but it is not required for reuse of the computer vision dataset.

## 2 Background

The dataset was compiled in the context of research on the computational representation of physical board games. While many studies on game AI and computer vision rely on synthetic or digitally generated environments, fewer datasets are derived from games played on physical boards, where visual perception and symbolic state representation must be aligned. This work focuses on data generation from real-world tabletop gameplay to support the study of image-based board state extraction and structured game representations.

Connect Four was selected as a controlled, deterministic two-player game with a fixed board structure and well-defined rules [2], making it suitable for mapping visual observations and symbolic board states. Human-versus-human games played on a physical board were recorded to capture realistic game-play conditions, including variations in lighting, camera perspective, and piece placement. The methodological background includes frame-level video annotation, object-based board and piece labeling, and rule-based conversion of visual annotations into structured board matrices and vectorized representations. As an auxiliary downstream resource, a large set of playable board states was also generated computationally with optimal move labels using established game-solving methods. This component is intended to support later decision-making stages once visual state extraction has been completed.

## 3 Data Description

The dataset consists of five interlinked datasets derived from the recorded Connect Four gameplay. The components include raw video recordings, annotated image frames, vectorized board representations, and an auxiliary optimal-move dataset. All files are provided in standard formats (MP4, JPG, TXT, and CSV) and are accompanied by metadata files. The following subsections describe the structure, content, and file organization of each dataset component.

The dataset includes >50 unique object instances in the form of distinct physical board states observed across 52 complete human-played games and several hundred annotated video frames. Board regions, disc instances (red and green), and empty cells are represented across well over 20 distinct images per class, without the use of data augmentation. The recordings capture natural variability in lighting conditions, camera viewpoints, hand positions, partial occlusions, and board orientation arising from real-world gameplay. Table 1 shows an overview of the dataset.

**Table 1:** Overview of the datasets and tools included in the Connect Four dataset.

Dataset / Tool	Description	File Type	Entries	Key Fields
Video dataset	Contains full Connect Four games ending in win, loss and draw	MP4	52	No annotations
Board Annotated Dataset	Boards marked for object detection	JPG, TXT	367	image_id; game_id; turn; matrix[6][7]
Piece Annotated Dataset	Coordinates and color of each disc	JPG, TXT	251	image_id; cell_id; x; y; color
Game Image Vectorized Dataset	Flattened 42-element vectors of board states	JPG, TXT	1073	image_id; vector[42]
Optimal Move Dataset	Board vectors with optimal move labels	CSV	1000,000	vector[42]; optimal_move (0–6)

### 3.1 VIDEO dataset

The video dataset consists of 52 Connect Four game recordings in MP4 format. Videos 1–40 had a resolution of 720×1280, whereas the remaining videos had a resolution of 1080×1920. The shortest video duration is 7.76 s, the longest is 263.92 s, and the average duration is 75.2 s. Metadata, including the file name, resolution, and duration, is provided in the metadata.csv file. Table 2 shows the sample metadata entries.

**Table 2:** Sample metadata of the videos from metadata.csv.

File Name	Width	Height	Duration (s)
38.mp4	720	1280	90.8
39.mp4	720	1280	90.85
4.mp4	720	1280	37.52
40.mp4	720	1280	19.84
41.mp4	1080	1920	25.97
42.mp4	1080	1920	16.43

### 3.2 Board annotated dataset

The board annotated dataset contains labeled Connect Four board images with bounding boxes drawn around the board. Each annotation specifies the location and class of the board using YOLO format. Each line in the annotation file follows the structure described below.

**Fig. 1:**
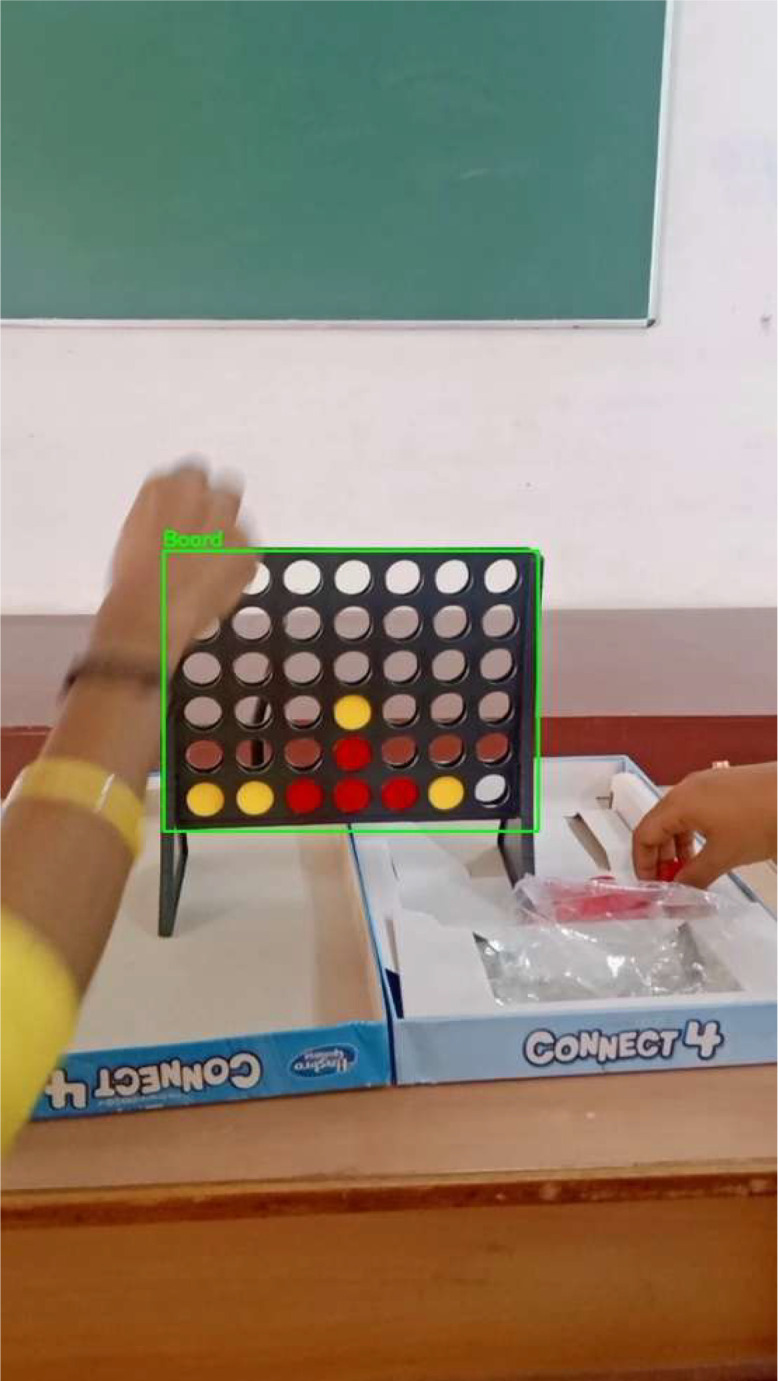
Annotated Connect Four board image showing the bounding boxes around the board positions.

< class_id > 〈x_center〉 〈y_center〉 〈width〉 〈height〉.

where all the coordinates are normalized between 0 and 1. The dataset includes 367 annotated frames derived from gameplay videos. Fig. 1. shows an example of an annotated image of a board.

### 3.3 PIECE annotated dataset

Each image contains bounding boxes around red discs, yellow discs, and active empty cells on the game board. Discs located outside the board area may appear in some frames but are not annotated in the dataset. The dataset includes 7256 instances of empty cells, 1697 instances of red discs, and 1589 instances of yellow discs. Each annotation followed the YOLO format.

**Fig. 2:**
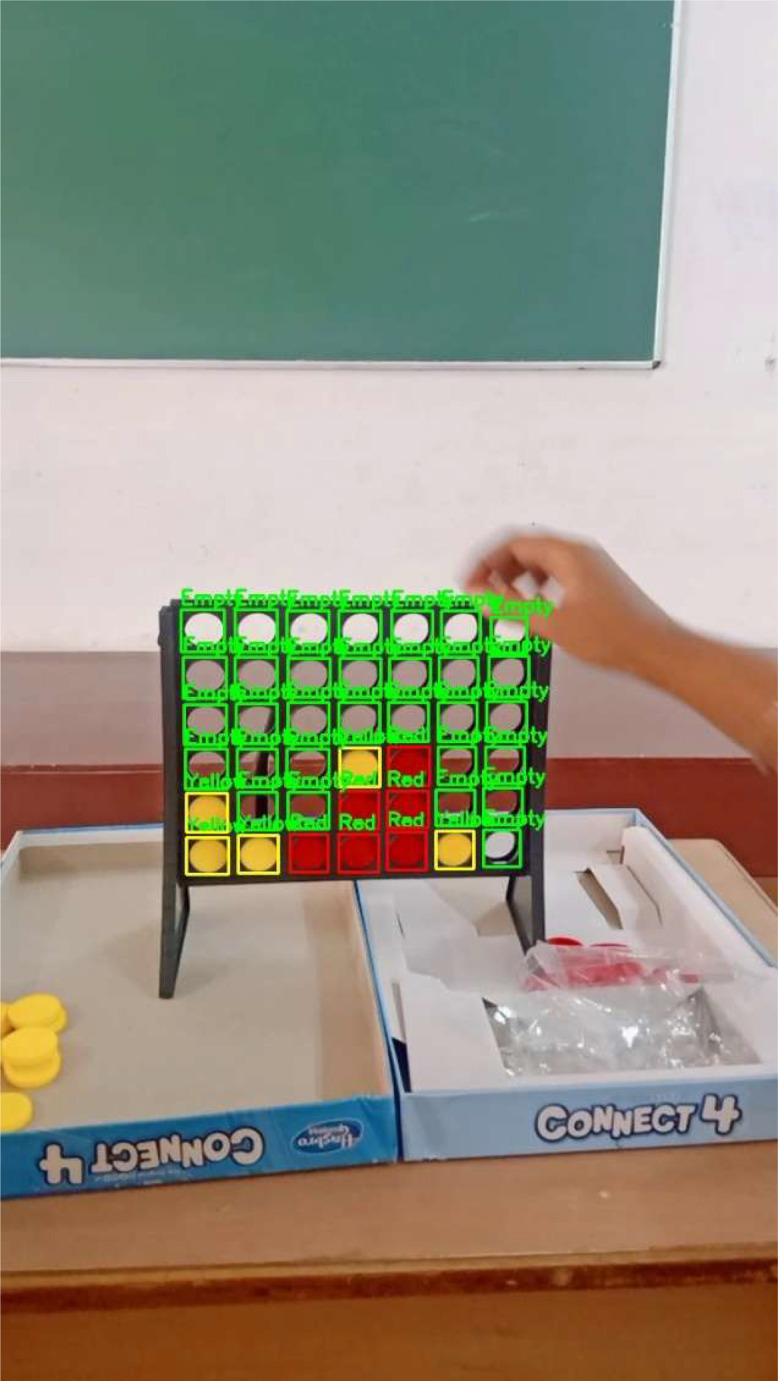
Example of annotated Connect Four pieces showing bounding boxes for red, yellow, and empty cells.

< class_id > 〈x_center〉 〈y_center〉 〈width〉 〈height〉.

where all values are normalized between 0 and 1. Fig 2 shows an example of an annotated image highlighting the detected pieces.

### 3.4 Game image vectorized dataset

The game image vectorized dataset consists of selected frames extracted from the gameplay videos. Each frame was named sequentially (e.g., frame0001.jpg, frame0002.jpg) and paired with a corresponding text file containing the matrix representation. The images were stored in .jpg format, and the associated vector files are stored in .txt format using the following structure.

0000,000

0000,000

0000,000

000YR00

00RYR00

Y0YRYRR

Here, `0' indicates an empty cell, whereas `R' and `B' represent red and blue discs, respectively. Each image–text pair provides a direct mapping between the visual configuration and the corresponding structured board state.

The details about the dataset are available in metadata.csv. Table 3 presents a sample entry from metadata.csv. The metadata file reports the number of annotations per folder. The dataset contains 52 folders with a total of 1073 annotations, with file counts ranging from 7 to 44 annotations.

**Table 3:** Sample metadata of the game image vectorized dataset from metadata.csv.

File Name	No of Annotations
19	21
2	33
20	15
21	21
22	13
23	29

### 3.5 Auxillary optimal MOVE dataset

The auxiliary dataset contains one million unique Connect Four board configurations stored in CSV format. Each record includes a unique identifier, player-to-move information, move sequence, move count, flattened board representation, and corresponding move labels generated using different methods. This dataset is provided separately from the video recordings and visual annotations. Sample entries are shown in Table 4.

**Table 4:** Example instance from the best move dataset.

Column	Instances
**id**	ff120f047f40c4fc94c3b6c601695b77
**player_to_move**	1
**move_sequence**	1521,542,433,453,163
**move_count**	16
**board_flat**	000,000,000,000,000,002,000,020,112,002,122,100,111,221
**velena_strong**	0
**3ply**	0
**5ply**	4
**7ply**	0
**9ply**	6

Example instance from the Best Move Dataset. Each record included the following fields:

• **id**: A unique identifier generated using a hash function.
• **player_to_move**: The player who is to make the next move (possible values: 1 or 2).
• **move_sequence**: The sequence of columns played by each player, ranging from 0 to 6.
• **move_count**: Number of moves in the sequence.
• **board_flat**: A flattened array representation of the board.
• **velena_strong**: The optimal move ranging from −1 to 6 predicted by the Velena engine [3], a knowledge-based Connect Four engine. −1 indicates that Velena was unable to predict the optimal move.
• **3_ply, 5_ply, 7_ply, 9_ply**: Optimal moves predicted by the minimax algorithm [4] at the respective search depths.

In the dataset, there are 211,857 early move positions, 635,726 mid-move positions, and 211,398 late-move positions, classified solely based on the number of moves made.

## 4 Experimental Design, Materials and Methods

**Fig. 3:**
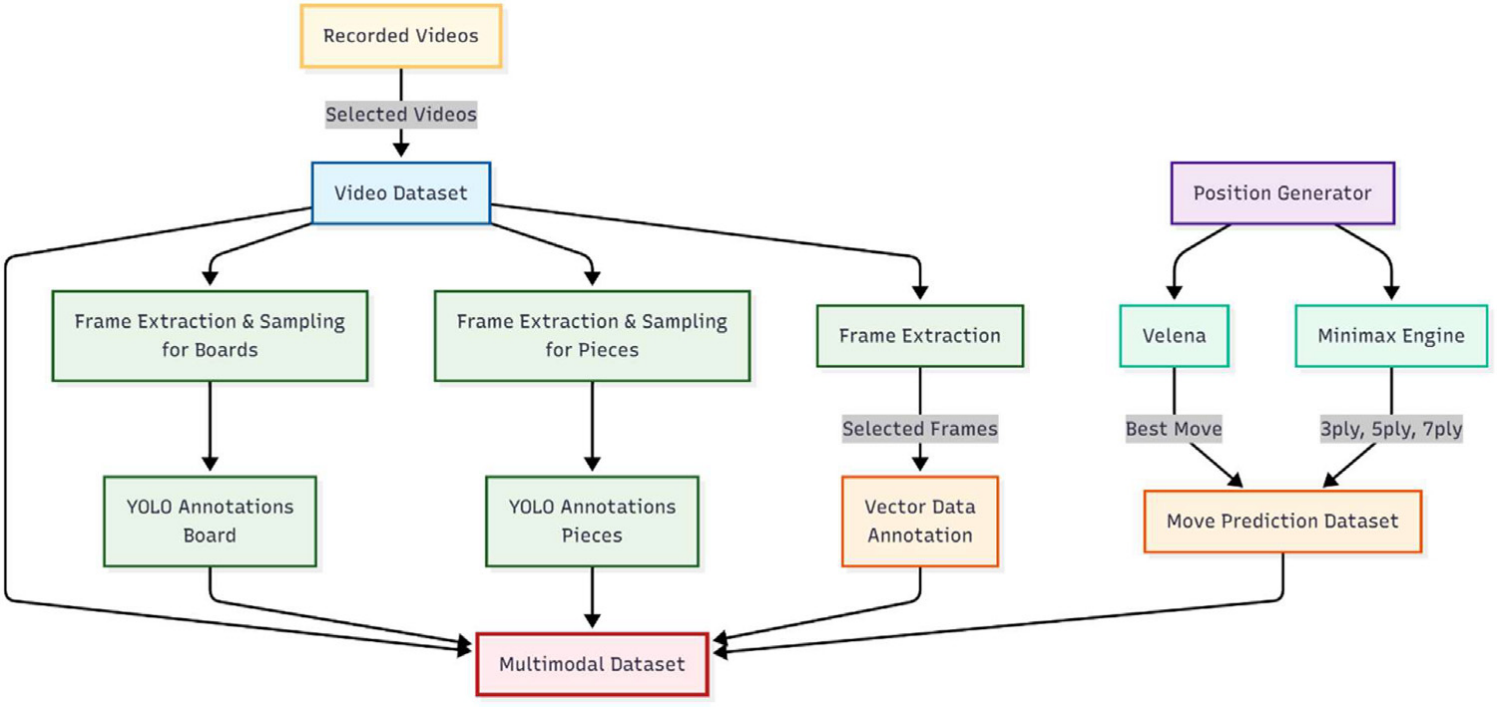
Overview of the dataset generation pipeline.

The dataset was generated through a structured pipeline consisting of two primary data sources: (1) datasets derived from human-played Connect Four games recorded on a physical board and (2) synthetically generated gameplay data used to compute the optimal move labels. The overall dataset generation pipeline is shown in Fig. 3. The human-played branch includes video acquisition, frame extraction, and visual annotation, while the synthetic branch involves computational generation of playable board states and corresponding move labels.

### 4.1 VIDEO dataset acquisition

A total of 20 volunteers were randomly paired to play Connect Four games on a physical board. Gameplay was recorded using smartphone cameras positioned to capture the full board area. In total, 93 video recordings were collected. From these, 52 videos were selected for inclusion based on visibility of the board, completeness of gameplay, and absence of severe occlusions or recording interruptions. These selected videos form the basis for subsequent frame extraction and annotation.

### 4.2 BOARD annotation procedure

From the selected gameplay videos, 371 image frames were extracted for board-level annotation. Board regions were annotated for object detection using the Label Studio [5] annotation tool. Each annotation specifies the bounding box corresponding to the game board within the image frame. All annotated frames were manually reviewed to ensure consistency in labeling and alignment with the physical boundaries of the board.

### 4.3 PIECE annotation procedure

Piece-level annotations were created using Label Studio for object detection. Instead of annotating the entire board region, individual game elements were labeled, including red discs, yellow discs, and active empty cells within the board area. Some frames contain discs located outside the board region; these elements were not annotated. Only discs and empty cells that were part of the playable board state were included in the annotations. All annotations were stored in text files using normalized bounding box coordinates consistent with the object detection format.

### 4.4 GAME image vectorization procedure

The game image vectorized dataset was generated using a custom annotation tool developed to convert gameplay frames into structured board representations. Each selected video was first divided into individual frames. Frames were displayed sequentially within the annotation interface, allowing annotations to be created or updated for each frame. When an annotation was saved, the corresponding image file and its associated board representation were stored as a paired record.

The tool supports three frame extraction modes: all frames, uniform sampling at a user-defined interval, and keyframe extraction. Additional functions include filtering to display only annotated frames and options to move processed frames and annotation files into separate folders before loading subsequent videos.

**Fig. 4:**
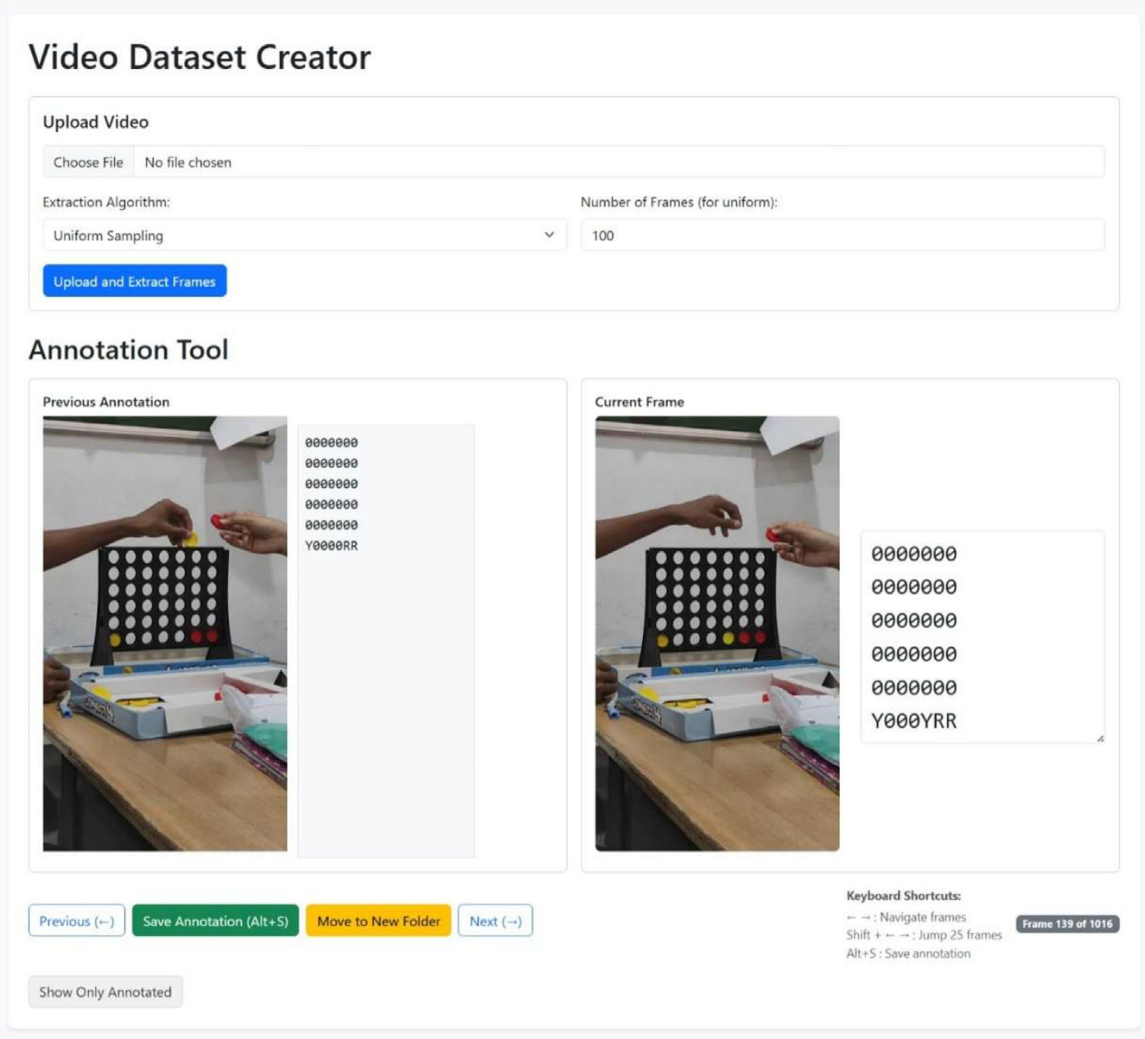
Custom annotation tool used for creating vectorized board representations from Connect Four gameplay frames.

Fig. 4 shows the annotation tool interface used during the vectorization process. The tool was implemented in Python, using OpenCV and NumPy for image processing and Flask, HTML, and JavaScript for the graphical user interface. The tool outputs synchronized image–vector pairs that represent the board state corresponding to each annotated frame.

### 4.5 AUXILIARY optimal MOVE dataset generator

The auxiliary optimal move dataset was generated by producing one million unique Connect Four board positions through random self-play. During generation, each board state was encoded and checked for uniqueness using a hash-based comparison. If a generated board configuration already existed in the dataset, it was discarded, and a new position was generated. To ensure that only valid game states were retained, each move sequence was validated after every move to confirm that further play was possible; sequences that reached invalid or terminal configurations prematurely were regenerated.

Move labels were computed for each board position using the Velena engine. For positions where the engine was unable to return a move, the corresponding move label was set to −1. In addition, move labels were computed using the minimax algorithm at search depths of 3, 5, 7, and 9 ply. When multiple legal moves received identical evaluation scores at a given depth, one move was selected at random and recorded in the dataset.

## Limitations

The gameplay videos and visual annotations were collected using multiple physical Connect Four boards from a single commercial brand. As a result, visual properties such as board texture, color tone, disc appearance, grid geometry, and material finish are consistent across recordings and may not reflect the full range of visual variation present across different brands or board designs.

Although the dataset includes recordings captured under varying lighting conditions, camera viewpoints, hand positions, and partial occlusions, the use of boards from a single brand limits variability arising from differences in manufacturing, materials, and design found across other commercially available boards.

The auxiliary optimal-move dataset is generated independently of the gameplay videos through computational self-play. While uniqueness and playability checks were applied, the distribution of board states in this auxiliary dataset may differ from those observed in human-played games.

For some board states in the auxiliary dataset, multiple legal moves receive identical evaluation scores. In such cases, a single representative move is recorded based on engine behavior or random selection at a given search depth, and alternative equivalent moves re not explicitly stored.

## Ethics Statement

The authors declare that the data collection involved voluntary participation in board game play and did not include any personal, sensitive, or identifiable information. Informed consent was obtained from all participants before video recording. The authors have read and comply with the ethical requirements for publication in *Data in Brief*.

## Declaration of generative AI and AI-assisted technologies in the manuscript preparation process

During the preparation of this work the author(s) used ChatGPT in order to paraphrase statements. After using this tool/service, the author(s) reviewed and edited the content as needed and take(s) full responsibility for the content of the published article.

## CRediT authorship contribution statement

**Pankaj Kumar G:** Conceptualization, Methodology, Software, Validation, Formal analysis, Investigation, Resources, Data curation, Writing – original draft, Writing – review & editing, Visualization, Project administration. **Anitha M L:** Conceptualization, Methodology, Validation, Investigation, Resources, Data curation, Writing – original draft, Writing – review & editing, Supervision. **Arun Kumar M N:** Conceptualization, Methodology, Validation, Investigation, Resources, Data curation, Writing – original draft, Writing – review & editing, Supervision.

## Data Availability

ZenodoMultimodal Dataset for Connect Four (Original data).
